# Amphotericin B Specifically Induces the Two-Component System LnrJK: Development of a Novel Whole-Cell Biosensor for the Detection of Amphotericin-Like Polyenes

**DOI:** 10.3389/fmicb.2020.02022

**Published:** 2020-08-21

**Authors:** Ainhoa Revilla-Guarinos, Franziska Dürr, Philipp F. Popp, Maximilian Döring, Thorsten Mascher

**Affiliations:** Department of General Microbiology, Institut für Mikrobiologie, Technische Universität Dresden, Dresden, Germany

**Keywords:** amphotericin, antifungal polyenes, drug discovery, fungal infections, nystatin, stress response, two-component system, whole-cell biosensor

## Abstract

The rise of drug-resistant fungal pathogens urges for the development of new tools for the discovery of novel antifungal compounds. Polyene antibiotics are potent agents against fungal infections in humans and animals. They inhibit the growth of fungal cells by binding to sterols in the cytoplasmic membrane that subsequently causes pore formation and eventually results in cell death. Many polyenes are produced by Streptomycetes and released into the soil environment, where they can then target fungal hyphae. While not antibacterial, these compounds could nevertheless be also perceived by bacteria sharing the same habitat and serve as signaling molecules. We therefore addressed the question of how polyenes such as amphotericin B are perceived by the soil bacterium, *Bacillus subtilis*. Global transcriptional profiling identified a very narrow and specific response, primarily resulting in strong upregulation of the *lnrLMN* operon, encoding an ABC transporter previously associated with linearmycin resistance. Its strong and specific induction prompted a detailed analysis of the *lnrL* promoter element and its regulation. We demonstrate that the amphotericin response strictly depends on the two-component system LnrJK and that the target of LnrK-dependent gene regulation, the *lnrLMN operon*, negatively affects LnrJK-dependent signal transduction. Based on this knowledge, we developed a novel whole-cell biosensor, based on a P_*lnrL*_-*lux* fusion reporter construct in a *lnrLMN* deletion mutant background. This highly sensitive and dynamic biosensor is ready to be applied for the discovery or characterization of novel amphotericin-like polyenes, hopefully helping to increase the repertoire of antimycotic and antiparasitic polyenes available to treat human and animal infections.

## Introduction

Fungal infections are a major threat to human health: close to one billion patients suffer annually from various types of mycotic diseases, such as mild skin and nail infections. When associated with immunodeficiency disorders, such fungal infections can lead to severe medical complications and in serious cases have fatal consequences (Bongomin et al., 2017). The treatment of fungal infections is difficult: only few compound classes have been approved for antifungal therapy, and even their application is restricted due to issues with regard to their toxicity, or fungistatic versus fungicidal action (Aldholmi et al., 2019). This generates a major threat for the rise of antifungal-resistant fungal pathogens (Revie et al., 2018; Geddes-McAlister and Shapiro, 2019). Thus, there is a clear need for identifying new compounds for antifungal treatment (Almeida et al., 2019).

**FIGURE 1:**
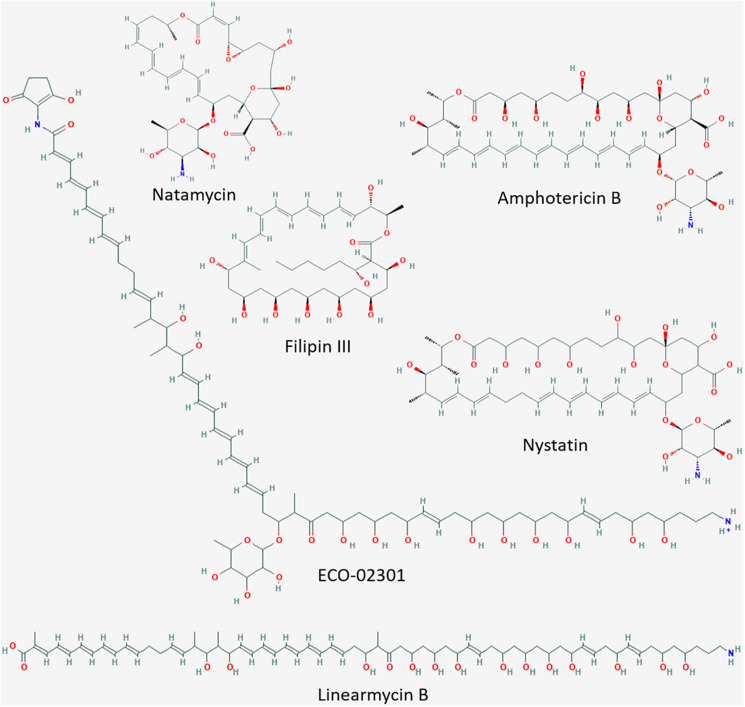
Cyclic and linear polyenes. Molecular structure images downloaded from National Center for Biotechnology Information, PubChem Database (accessed on Apr. 20, 2020) (Kim et al., 2015). Amphotericin B, CID = 5280965 (https://pubchem.ncbi.nlm.nih.gov/compound/5280965#section~=~2D-Structure); nystatin, CID = 6433272 (https://pubchem.ncbi.nlm.nih.gov/compound/6433272#section~=~2D-Structure); ECO-02301, CID = 90658124 (https://pubchem.ncbi.nlm.nih.gov/compound/90658124#section = 2D-Structure); filipin III, CID = 6433194 (https://pubchem.ncbi.nlm.nih.gov/compound/6433194#section = 2D-Structure); natamycin, CID = 5284447 (https://pubchem.ncbi.nlm.nih.gov/compound/5284447#section = 2D-Structure); linearmycin B, CID = 10328913 (https://pubchem.ncbi.nlm.nih.gov/compound/10328913#section = 2D-Structure).

Natural polyenes are a primary choice for the treatment of fungal infections. Hence, the identification of novel polyenes or their modification poses an ideal approach to develop novel potent antifungal drugs (Rochette et al., 2003; Zotchev, 2003; Madden et al., 2014). As their name indicates, polyenes are poly unsaturated (at least three alternating double bonds), cyclic or linear organic compounds (Figure 1). They are classified by the number of conjugated double bonds as trienes, tetraenes, pentaenes, etc., and include a variety of chemical structures with different biological activities (Madden et al., 2014).

Cyclic polyenes belonging to the macrolide family, such as amphotericin B, candicidin, nystatin, and natamycin (Figure 1), contain a large macrolactone ring and have received considerable attention since many of them are used in human as well as animal therapy (Hamilton-Miller, 1973; Rochette et al., 2003; Zotchev, 2003). The polyene macrolide amphotericin B, produced by *Streptomyces nodosus* ATCC14899, is among the most commonly applied antifungal therapies (Caffrey et al., 2001; Cereghetti and Carreira, 2006). Amphotericin B forms trans-membrane channels in sterol-containing membranes. Its affinity (and hence activity) is higher toward ergosterol (characteristic for fungal membranes) than toward cholesterol (present in mammalian membranes) (Baginski et al., 2005). Due to this selectivity, amphotericin B is the most important antibiotic for the treatment of life-threatening systemic mycotic infections in humans (Torrado et al., 2008). Amphotericin B, alone or in combination with other drugs, is the only well-stablished therapy to treat primary amebic meningoencephalitis (PAM) caused by the free-living ameba *Naegleria fowleri* (Grace et al., 2015). In addition, it is also used for the treatment of visceral leishmaniasis (VL) caused by protozoan parasites of the genus *Leishmania* (Chattopadhyay and Jafurulla, 2011). Unfortunately, treatment of these, often fatal, systemic infections usually requires high dosages of amphotericin B, resulting in adverse side reactions in the human body such as nephrotoxicity, shaking chills, fever and anemia (Gallis et al., 1990; Zotchev, 2003; Antillón et al., 2016). Consequently, new amphotericin-like polyenes with less severe side effects for humans and stronger antimycotic and antiparasitic activities are highly sought after as alternatives to the conventional treatments (Tevyashova et al., 2013; Antillón et al., 2016).

Research efforts suggest three different directions: a) the creation of new derivatives from existing polyenes like amphotericin B (Power et al., 2008; Debnath et al., 2012; Tevyashova et al., 2013); b) new formulations for drug delivery aimed at decreased toxicity (Hamill, 2013; Palma et al., 2018); and c) the identification of new linear or cyclic polyenes from natural producers (Cai et al., 2007; Stodulkova et al., 2011; Vartak et al., 2014; Wang et al., 2017; Yao et al., 2018). The latter strategy can be accomplished by harnessing the metabolic diversity of Streptomycetes, which are known to produce a vast variety of natural products including many natural polyenes (Baltz, 2008; Madden et al., 2014). In order to efficiently identify these natural compounds, bacterial whole-cell biosensors have proven to be powerful screening tools (Wolf and Mascher, 2016).

Whole-cell biosensors consist of modified bacterial reporter strains, in which an antibiotic- specific promoter is transcriptionally fused to a reporter gene/operon. The selected promoters should be tightly switched off in the absence of the inducer but activated in a dose response manner in its presence, at concentrations well below the minimal inhibitory concentration (MIC) of the biosensor strain (Wolf and Mascher, 2016). The promoter selectivity can either be determined by the antibiotic target (e.g., inhibition of a specific essential cellular process) or by its chemical nature (Hutter et al., 2004; Urban et al., 2007). The Gram-positive model organism *Bacillus subtilis* has been widely used for developing whole-cell biosensors due to its GRAS status, wide availability of known antibiotic-inducible promoters and its ease of genetic manipulations (Hutter et al., 2004; Urban et al., 2007; Czarny et al., 2014).

In the soil microbiome where *B. subtilis* and *Streptomyces* spp. coexist, *B. subtilis* is exposed to the diversity of *Streptomyces*-produced antibiotics at fluctuating concentrations. Beyond the inhibitory action of antibiotics and the corresponding stress responses against them, hormesis has also been observed, which describes a concentration-dependent transcription modulation of antibiotics independent of the antimicrobial activity. Antibiotics with stimulatory effects at subinhibitory concentrations are proposed to act as cell-signaling molecules involved in modulating the interactions within microbial populations (Yim et al., 2007). In this work, we explore such natural soil-microbiome interactions and the effect of the presence of bioactive microbial metabolites. This allowed us to identify new biosensor candidates for expanding the tools for screening novel antibiotics, such as antifungals. Challenging *B. subtilis* with the antifungal amphotericin B resulted in the induction of the *lnrLMN* operon encoding an ABC transporter that is regulated by the LnrJK two-component signaling system (TCS). We characterized the *lnrL* promoter and the signal transduction mediated by the LnrJK TCS to develop a whole-cell biosensor specific for amphotericin-like polyenes in *B. subtilis*. We removed the negative regulatory constrains exerted by the LnrLMN transporter on LnrJK-dependent signaling to implement a biosensor with optimized promoter readout and enhanced dynamics. We demonstrate the robustness and sensitivity of our novel biosensor with different samples. Our data highlights the potential of exploring bacterial perception of harmless bioactive molecules present in their natural niches and it indicates that this cell-based biosensor could be very useful for identifying new amphotericin-like polyene antibiotics with possible therapeutic applications.

## Materials and Methods

### Bacterial Strains, Plasmids and Growth Conditions

Table 1 lists the strains used in this study. *Escherichia coli* DH10β was used as an intermediate host for cloning. *B. subtilis* and *E. coli* cells were routinely grown in Luria-Bertani medium (LB-Medium (Luria/Miller), Carl Roth GmbH + Co., KG, Karlsruhe, Germany) at 37°C with agitation. 1.5% (w/v) agar (Agar-Agar Kobe I, Carl Roth GmbH + Co., KG, Karlsruhe, Germany) was added to prepare the corresponding solid media. *Streptomyces* spp. were grown in MYM medium supplemented with trace elements [0.4% (w/v) maltose, 0.4% (w/v) yeast extract, 1% (w/v) malt extract, trace elements: 0.004% (w/v) ZnCl_2_, 0.02% (w/v) FeCl_3_ × 6H_2_O, 0.001% (w/v) CuCl_2_ × 2H_2_O, 0.001% (w/v) MnCl_2_ × 4H_2_O, 0.001% (w/v) Na_2_B_4_O_7_ × 10H_2_O, 0.001% (w/v) (NH_4_)_6_Mo_7_O_24_ × 4H_2_O (w/v)] and for solid medium 2% (w/v) agar was added. *Streptomyces* spp. strains were either incubated at 28°C or at room temperature. All bacterial strains were stored at −80°C in their corresponding growth media supplemented with 20% (v/v) glycerol (Carl Roth GmbH + Co., KG, Karlsruhe, Germany). Ampicillin (Carl Roth GmbH + Co., KG, Karslruhe, Germany) 100 μg ml^–1^ was added to *E. coli* when required. Chloramphenicol (Sigma-Aldrich, Merck KGaA, Darmstadt, Germany) 5 μg ml^–1^, kanamycin (VWR International GmbH, Darmstadt, Germany) 10 μg ml^–1^ or erythromycin (Sigma-Aldrich, Merck KGaA, Darmstadt, Germany) 1 μg ml^–1^ and lincomycin (Sigma-Aldrich, Merck KGaA, Darmstadt, Germany) 25 μg ml^–1^ (MLS resistance) were added to *B. subtilis* when required.

**TABLE 1:** Bacterial strains used in this study.

Strain	Description^a^	Source or references
*Escherichia coli* DH10β	F^–^ *mcrA* Δ(*mrr-hsdRMS-mcrBC*) Φ80d*lacZ*Δ*M15* Δ*lacX74 endA1 recA1 deoR* Δ(*ara,leu*)7697 *araD139 galU galK nupG rpsL* λ^–^	Laboratory stock
*Streptomyces nodosus* ATCC14899	−	DSMZ – German Collection of Microorganisms and Cell Cultures
*Streptomyces noursei*	−	Laboratory stock
*Bacillus subtilis*		
W168	Wild type; *trpC2*	Laboratory stock
TMB1151	W168 Δ*liaIH*	Radeck et al., 2016
TMB3822	W168 pBS3C*lux*-P_*liaI*_	Popp et al., 2017
TMB4173 (P_*lnrL*127_)	W168 *sacA*:*cm*^r^ P_*lnrL*_ (127)- *luxABCDE*	This study
TMB4220 (P_*lnrL*468_)	W168 *sacA*:*cm*^r^ P_*lnrL*_ (468)- *luxABCDE*	This study
TMB4221 (P_*lnrL*383_)	W168 *sacA*:*cm*^r^ P_*lnrL*_ (383)- *luxABCDE*	This study
TMB4222 (P_*lnrL*308_)	W168 *sacA*:*cm*^r^ P_*lnrL*_ (308)- *luxABCDE*	This study
TMB4223 (P_*lnrL*231_)	W168 *sacA*:*cm*^r^ P_*lnrL*_ (231)- *luxABCDE*	This study
TMB4237	W168 *lnrLMN*:*kan*^r^	This study
TMB4238	W168 *lnrJK*:*kan*^r^	This study
TMB4241	W168 Δ*liaIH lnrLMN*:*kan*^r^	This study
TMB5408 (P_*lnrL*147_)	W168 *sacA*:*cm*^r^ P_*lnrL*_ (147)- *luxABCDE*	This study
MB5422	W168 *lnrJK*:*kan*^r^, *sacA*:*cm*^r^ P_*lnrL*__231_-*luxABCDE*	This study
TMB5423	W168 *lnrLMN*:*kan*^r^, *sacA*:*cm*^r^ P_*lnrL*__231_-*luxABCDE*	This study
TMB5473 (P_*lnrL*191_)	W168 *sacA*:*cm*^r^ P*lnrL* (191<-)- *luxABCDE*	This study
TMB5578	W168 Δ*lnrLMN*, clean deletion	This study
TMB5600	W168 Δ*lnrLMN sacA*:*cm^r^ P_*lnrL*231_-luxABCDE*	This study
TMB5771	W168 *yhbIJ-yhcABC*:*kan*^r^	This study
TMB5772	W168 *mdtRP*:*mls*^r^	This study
TMB5779	W168 *sacA*:*cm^r^ P_yhbI_ luxABCDE*	This study
TMB5780	W168 *sacA*:*cm^r^ P_mdtR_ luxABCDE*	This study

*^a^cm^r^, chloramphenicol resistance; kan^r^, kanamycin resistance; mls^r^, macrolide-lincosamide-streptogramine resistance.*

### Cloning Procedures

#### Construction of Transcriptional Promoter-*luxABCDE* Fusions

All vectors and plasmids used in this study are listed in Table 2; all oligonucleotides used in this study are listed in Supplementary Table S1. Ectopic integrations of the different promoter-*luxABCDE* fusion fragments into the *B. subtilis sacA* locus were constructed based on the vector pBS3C-*lux* (Radeck et al., 2013). Promoter fragments were generated by PCR from genomic DNA with specific primers (Supplementary Table S1) designed according to the BioBrick cloning standard (Radeck et al., 2013). For the P_*lnrL*468_ promoter, primers TM5098 and TM5695 were used; for all 5′ end truncations, the reverse primer TM5695 was used in combination with the corresponding forward primers; for the 191 bps length 3′ end truncation, the forward primer TM5789 was used in combination with TM6014 (Supplementary Table S1). For the confirmation of the RNA seq results, two additional promoter-*luxABCDE* fusions - P_*yhbI*_ and P_*mdtR*_ - were constructed using the primer pairs TM6334/6335 and TM6336/6337. After transformation into *E. coli* DH10β, ampicillin resistant colonies were examined by PCR with primers TM2262/2263, the inserts were verified by DNA sequencing and the resulting pBS3C-derived plasmids (Table 2) were linearized with *Sca*I and used to transform *B. subtilis*. Correct integration into the *sacA* locus was checked by amplification of an *up-* and *down-* PCR fragment with primers TM2505/2506 and TM5955/5956 or TM2507/2508, respectively. In each case, two independent positive clones were selected as reporter strains.

**TABLE 2:** Vectors and plasmids used in this study.

Name	Description (primers used for cloning/antibiotic resistances^a^)	Source or references
**Vectors**		
*pBS3Clux*	*pAH328* derivative; *amp*^*r*^, *cm*^*r*^, *sacA*’…’*sacA*, *luxABCDE*	Laboratory stock
*pMAD*	*erm*^r^, ori(pE194-Ts), MCS-P*_clpB–bgaB_*, ori(pBR322), *bla*^r^	Arnaud et al., 2004
*pDG647*	*pSB119*, *erm*^r^	Guérout-Fleury et al., 1995
*pDG783*	*pSB118*, *kan*^r^	Guérout-Fleury et al., 1995
**Plasmids**		
*pBS3C*-P_*lnrL*_(468)-*lux*	TM5098/TM5695; *cm^r^, amp^r^*	This study
*pBS3C*-P_*lnrL*_(383)-*lux*	TM5787/TM5695; *cm^r^, amp^r^*	This study
*pBS3C*-P_*lnrL*_(308)-*lux*	TM5788/TM5695; *cm^r^, amp^r^*	This study
*pBS3C*-P_*lnrL*_(231)-*lux*	TM5789/TM5695; *cm^r^, amp^r^*	This study
*pBS3C*-P_*lnrL*_(147)-*lux*	TM5855/TM5695; *cm^r^, amp^r^*	This study
*pBS3C*-P_*lnrL*_(127)-*lux*	TM5694/TM5695; *cm^r^, amp^r^*	This study
*pBS3C*-P_*lnrL*_(191<-)-*lux*	TM5789/TM6014; *cm^r^, amp^r^*	This study
*pBS3C*-P_*yhbI*_-*lux*	TM6334/TM6335; *cm^r^, amp^r^*	This study
*pBS3C*-P_*mdtR*_-*lux*	TM6336/TM6337; *cm^r^, amp^r^*	This study

*^a^cm^r^, chloramphenicol resistance, amp^r^, ampicillin resistance, erm^r^, erythromycin resistance, kan^r^, kanamycin resistance, bla^r^, β-lactam resistance.*

#### Construction of Allelic Replacement Mutant Strains

Mutant strains lacking either *lnrJK* or *lnrLMN* (strains TMB4238 and TMB4237, respectively, Table 1) were generated by allelic replacement mutagenesis in *B. subtilis* W168 using long flanking homology (LFH)-PCR (Mascher et al., 2003). The genes were replaced by a kanamycin (*kan*) resistance cassette. The procedure was performed as described previously (Staroń et al., 2011). The same procedure was applied to construct the two *B. subtilis* W168 mutant strains *yhbIJ-yhcABC:kan* (TMB5771) and *mdtRP:mls* (TMB5772). Primer pairs used for amplification of the *kanamycin* and *mls* cassettes, up- and down-fragments, and primers used to check the allelic replacements are listed in Supplementary Table S1.

The reporter strains TMB5422 and TMB5423 (lacking *lnrJK* or *lnrLMN*, respectively) containing the P_*lnrL*231_-*luxABCDE* operon were created by transformation of TMB4238 and TMB4237 with pBS3C-P_*lnrL*_(231)-*lux* (Tables 1, 2).

#### Development of the Whole-Cell Biosensor

The *B. subtilis* Δ*lnrLMN* P_*lnrL*231_ whole-cell-biosensor (TMB5600, Table 1) was created by deleting the ABC-transporter *lnrLMN* and the subsequent introduction of the already created plasmid pBS3C-P_*lnrL*_(231)-*lux.* At first, two 1000 bp fragments up- and down of the *lnrLMN* operon were joined *in silico* (*lnrLMN*updo) and synthesized as gBlock by Integrated DNA Technologies (IDT, Coralville, IA, United States). The gBlock was solubilized according to manufacturer’s instructions and amplified using the primer pair TM6094/95. Afterward, the PCR product and the vector pMAD were digested with the restriction enzymes *Eco*RI and *Bam*HI (New England Biolabs, Ipswich, Massachusetts, United States) and ligated using the T4 DNA polymerase (New England Biolabs, Ipswich, MA, United States). The ligation mix was directly used for the transformation of *B. subtilis* W168 [see protocol for *B. subtilis* transformation (Harwood and Cutting, 1990)]. The pMAD based deletion was carried out as described in Arnaud et al. (2004). In brief, the *B. subtilis* transformants were selected on macrolide-lincosamide-streptogramines (MLS) LB agar plates that also contained X-gal at 30°C. Afterward, one blue colony was picked to inoculate an MLS-LB day culture. After an incubation of 2 h at 30°C, the temperature was shifted to 42°C for 6 h to induce an integration of the plasmid into the genome. The culture was plated again on MLS – X-gal LB agar plates and incubated at 45°C overnight. The successful integration of pMAD-*lnrLMNupdo* into the genome was checked by PCR using the primer pairs TM253/6026 and TM254/6027. Subsequently, positive clones were inoculated into an LB day culture without selection and incubated at 30°C for 6 h, then the temperature was shifted to 42°C again for 3h and finally the culture was plated on X-gal LB plates without selection and incubated at 42°C overnight. Positive clones, which did not show any β-galactosidase activity but MLS sensitivity, were examined by PCR with the primer pair TM6028/29 and successful deletion of *lnrLMN* was verified by subsequent sequencing (TM6028/6029/5694). Afterward, the strain W168 Δ*lnrLMN* (TMB5578, Table 1) was transformed with pBS3C-P_*lnrL*_(231)-*lux* to create the biosensor strain TMB5600.

### Reagents

The Amphotericin B solution (product code L0009) was purchased from Biowest (VWR International GmbH, Darmstadt, Germany). Filipin III (from *Streptomyces filipinensis*, product code F4767), natamycin (product code 32417), and sodium deoxycholate (product code D6750) were purchased from Sigma-Aldrich (Merck KGaA, Darmstadt, Germany). Nystatin (product code 15340029) was purchased from Gibco (Thermo Fisher Scientific, Waltham, Massachusetts, United States). Ethanol (product code 9065.4) and methanol (CP43.3) were purchased from Carl Roth (GmbH + Co., KG, Karslruhe, Germany).

### Preparation of *Streptomyces* spp. Subnatants

*Streptomyces* spp. subnatants were prepared by transferring *Streptomyces* spp. spore material into 20 ml liquid MYM medium in round culture dishes (ref. 633180; Greiner Bio-One International GmbH, Kremsmünster, Austria) and incubated at room temperature for 2–3 weeks. Afterward, the liquid MYM medium underneath the *Streptomyces* spp. cultures was transferred to a centrifugation tube and remaining cell particles were removed by centrifugation and sterile filtration (0.2 μm; Filtropur S 0.2, ref. 83.1826.001; Sarstedt AG & Co., KG, Nümbrecht, Germany). The resulting *Streptomyces* spp. subnatants were stored at 4°C until further use.

### Sensitivity Assays and Promoter Induction Assays in Liquid Medium

All the experiments were performed in Luria-Bertani medium. Overnight cultures of the strains under study were prepared with antibiotic selection when required. The day cultures (10 ml) were inoculated 1:1000 with overnight cultures and incubated at 37°C (220 rpm) without antibiotic selection until an OD_600_ of around 0.2 was reached. Then, the cell suspensions were diluted to an OD_600_ of 0.01, they were distributed into a 96-well plate (80 μl per well) and incubated at 37°C (continuous middle shacking) in the Synergy^TM^ NeoAlpha B plate reader (BioTek^®^, Winooski, VT, United States). After one hour of incubation, 20 μl of the substances under study (at 5 times the desired final concentration) were added to the wells and incubation at 37°C with continuous middle shaking was continued for further 18 h. For the sensitivity assays, the cells were plated in transparent 96-wells plates (ref. 83.3924, Sarstedt AG & Co., KG, Nümbrecht, Germany) and OD_600_ was measured every 5 min to monitor the growth rate. For the promoter induction assays, the cells were plated in black 96-wells plates (ref. 655097, Greiner Bio-One International GmbH, Kremsmünster, Austria) and besides OD_600_, luminesce was monitored every 5 min for at least 18 h.

In the case of amphotericin B whose composition includes sodium deoxycholate to improve solubility (in a ratio 250 μg/ml: 205 μg/ml, amphotericin B: sodium deoxycholate), a control sensitivity assay was performed with equivalent concentrations of pure deoxycholate. While some toxicity could be observed with the highest concentration of sodium deoxycholate (Supplementary Figure S1A), no promoter induction was detected (Supplementary Figure S1B) confirming the specific activation of P_*lnrL*_ by amphotericin B. In the case of filipin III and natamycin, control assays with the corresponding solvents, ethanol and methanol, respectively, performed with equivalent concentrations showed that the *B. subtilis* growth impairment was due to the solvent toxicity (Supplementary Figures S1C–F).

In the experiments with *Streptomyces sp.* subnatants, at concentrations ranging from 20% to 1.25%, addition of the suspensions caused a color change of the LB medium thus affecting the absorbance values. OD_600_ values were corrected by the mean blank values obtained from the different subnatants concentrations in LB medium without the inoculation with *B. subtilis*. In the case of the induction with pure compounds, the same correction was applied when necessary, otherwise the OD_600_ values were corrected by the mean blank values of LB medium prior to inoculation with cells. Afterward, the absolute luminescence values were divided by these corrected OD_600_ values resulting in relative luminescence units by OD_600_ (RLU/OD_600_).

### Spot-on-lawn Assay

The overnight and day cultures of the *B. subtilis* reporter strains were prepared in LB liquid medium as described above. In the case of the induction with *Streptomyces* spp. secreted natural products, *Streptomyces* spp. spore suspensions were spotted onto MYM agar plates and incubated for 3 days at room temperature or 28°C. Next, day cultures of the biosensor strain TMB5600 were incubated at 37°C (220 rpm) until an OD_600_ of around 0.4–0.7 was reached. Then, the cell suspensions were diluted to an OD_600_ of 0.01 in 10 ml of LB-soft (0.75%) agar, homogenized by shortly vortexing, and poured on top of the pre-grown *Streptomyces* spp. plates. In the case of induction with pure compounds, after a drying period of at least 10 min, the biosensor lawn was inoculated with 20 μl of macrolide polyene stock solutions [amphotericin B, 250 μg ml^–1^; nystatin, 3.33 mg ml^–1^, equivalent to 10^4^ U ml^–1^ (Lightbown et al., 1963)] on top of the agar. Subsequently, another drying period was conducted to allow the antibiotics to be completely absorbed into the agar. Afterward, all plates were incubated upside down overnight at 37°C. The luminescence output was measured with a FluorChem^TM^ SP from Alpha Innotech with an exposition time of 2 min at a high-medium intensity.

### RNA Sample Preparation and Sequencing

RNA-seq experiments were performed in triplicates with *B. subtilis* WT (BaSysBio) (Anagnostopoulos and Spizizen, 1961; Nicolas et al., 2012) in LB medium (Sigma L3522). Day cultures were inoculated from overnight cultures and grown until mid-exponential phase (OD_600_ approx. 0.4) at 37°C. Subsequently, a second day culture of 200 ml LB (Sigma L3522) was started with an OD_600_ = 0.1. Once this second day culture reached OD_600_ = 0.5, cells were split into 25 ml aliquots and either exposed to 10 μg ml^–1^ amphotericin B (final concentration) or remained untreated for 10 min. Even the highest amphotericin B concentration tested did not lead to a growth impairment (Supplementary Figure S2A). Therefore, a concentration of 10 μg ml^–1^ was selected ensuring full P_*lnrL*_ induction (Supplementary Figure S2C) and allowing a margin for potential expression of less sensitive amphotericin B responding genes, while preventing a big dilution of the RNA samples relative to the control samples. After treatment, cells were transferred to 50 ml falcons and growth was immediately stopped in an ice water bath followed by centrifugation at 8000 rpm at 4°C for 3 min. The supernatants were discarded, and the resulting pellets were stored at −80°C. RNA isolation was performed with a phenol-chloroform extraction method as previously described (Popp et al., 2020). The cDNA library was prepared using the NEB Ultra RNA directional prep kit for Illumina and sequencing was performed on an Illumina HiSeq3000 system. Sequencing reads were mapped to the BaSysBio 168 strain (NC_000964.3) using Bowtie2 (Langmead and Salzberg, 2012). The software program featureCounts of the Subread package (Liao et al., 2014) was applied to generate counts for known genes. Differentially expressed genes were identified using the R/Bioconductor package DESeq2 (Love et al., 2014). The raw and processed RNA sequencing data obtained in this study has been deposited at the NCBIs Gene Expression Omnibus (Edgar et al., 2002) and is accessible via the GEO accession number GSE148903.

## Results and Discussion

### Identification of the Amphotericin B Stimulon of *B. subtilis* by Transcriptome Profiling

*Streptomyces* spp. are common soil bacteria, where they produce antibiotics targeting fungi and bacteria (Baltz, 2008). However, at concentrations below inhibitory levels, antibiotics might also function as signaling molecules (Yim et al., 2007). As *B. subtilis* is also a soil inhabitant and competing for the same ecological niche, we aimed at identifying cellular processes activated in *B. subtilis* upon exposure to *Streptomyces*-produced bioactive molecules potentially present in the same habitat. We selected amphotericin B since it has only weak inhibitory activity toward bacteria and primarily targets fungal cells. We challenged *B. subtilis* wild type with a sublethal concentration of amphotericin B and compared the global RNA transcriptional profile with non-induced samples (for details see Materials and Methods).

**FIGURE 2:**
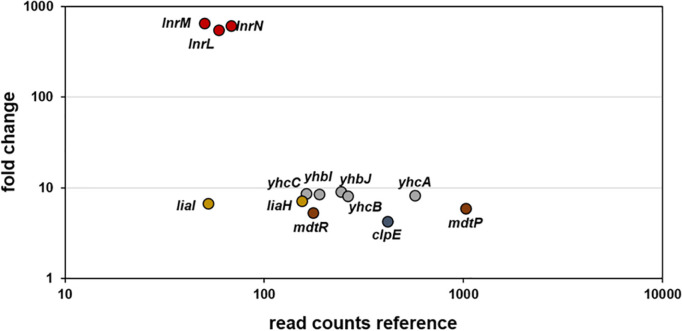
RNA-sequencing (RNA-seq) profile of *B. subtilis* upon amphotericin B treatment. Visualization of altered gene expression in *B. subtilis* upon 10 μg ml^–1^ amphotericin B exposure after 10 min, compared to non-induced control samples. Fold change of elevated RNA-seq counts are plotted over read counts in the reference condition. Same colored dots highlight genes encoded within an operon. For further details, see Table 3.

RNA-sequencing (RNA-seq) experiments were performed with *B. subtilis* treated with 10 μg ml^–1^ amphotericin B and gene expression profiles between treated and non-treated samples were compared 10 min post induction (see methods for details). Upon exposure to amphotericin B, only 13 genes were differentially expressed (Figure 2 and Table 3). Most prominent was the upregulation of the *lnrLMN* operon, an ABC transporter driven by the P_*lnrL*_ promoter and part of the LnrJK TCS regulon (Yamamoto et al., 1996). Previously, this system has been identified as resistance determinant against linearmycins (Stubbendieck and Straight, 2015). Additionally, moderate induction of the LiaRS TCS controlled genes *liaIH* was observed. This system is a specific marker for cell envelope stress and responds to interference with the lipid II cycle of cell wall biosynthesis (Mascher et al., 2004; Wolf et al., 2010). Further, amphotericin B treatment caused moderate induction of the *mdtRP* operon encoding a regulator and a multidrug efflux transporter involved in mediating resistance against several antibiotics (Kim et al., 2009). Induction of *clpE*, the ATPase subunit of the ClpE-ClpP protease, suggests a moderate general physiological impact upon amphotericin B exposure in *B. subtilis*. This protease controls the stability and activity of central transcriptional regulators, thereby influencing developmental decisions and stress-responses (Frees et al., 2007). Also, the *yhbIJ-yhcABC* operon showed moderate induction, however, so far only little is known about these genes and linking a physiological impact to amphotericin B response will require further investigations.

**TABLE 3:** RNA-seq results.

Gene(s)^1^	log2 fold change, *p*-value^2^	Regulators^3^	Products/function^3^
*lnr****LMN***	**9.33**, 5⋅10^–210^	SigG/F, LnrK	ABC transporter, resistance against linearmycin
*yhb****IJ****-yhc****ABC***	**3.17**, 4⋅10^–16^	−	Putative efflux system
*lia****IH***	**2.83**, 6⋅10^–4^	LiaRS	Phage shock protein, resistance against cell wall antibiotics
*mdt****RP***	**2.55**, 3⋅10^–38^	−	Multidrug-efflux system
***clpE***	**2.08**, 4⋅10^–6^	CtsR	ATPase subunit of ClpEP protease

*Differential gene expression between samples exposed to 10 μg ml^–1^ amphotericin B compared to non-induced conditions. Genes were considered that showed a log2 fold change greater than 2 and a p-value smaller than 0.05. No genes were significantly down regulated upon amphotericin B treatment. ^1^All genes with fold-induction ≥ 2 fold are indicated in bold. ^2^For each operon, the highest value is listed. ^3^(Putative) regulators and the function of the gene products are adapted from SubtiWiki (Zhu and Stülke, 2017).*

**FIGURE 3:**
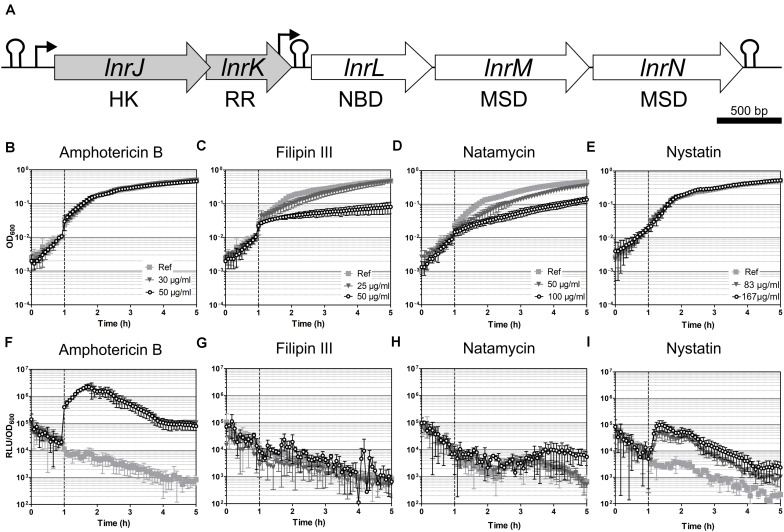
Induction spectrum of the LnrJKLMN system in *B. subtilis*. **(A)** Genetic organization of the *lnrJKLMN* operon (formerly known as *yfiJKLMN*) (Yamamoto et al., 1996). The genes *lnrJ* and *lnrK* (block gray arrows) encode for a two-component system; the genes *lnrL*, *lnrM* and *lnrN* (block white arrows) encode for an ABC transporter. Genes drawn to scale. Rho-independent transcriptional terminators are indicated by hairpins; promoters are indicated by black bent arrows; HK, histidine kinase; RR, response regulator; NBD, nucleotide binding domain; MSD, membrane spanning domain. **(B–I)** Induction of the P_*lnrL*468_ promoter by macrolide polyenes in liquid media. Effect of antibiotic exposure on growth is indicated as OD_600_ **(B–E)**, and promoter induction as relative luminescence units by OD_600_ **(F–I)**. The time of antibiotic addition is indicated with a vertical dashed line. The antibiotic concentrations used are indicated in the upper panel graphs. The results presented from **(B)** to **(I)** correspond to strain TMB4220. The experiments were performed at least in triplicate with two independent clones. Means and standard deviations are depicted.

In order to verify the RNA-seq data for the induced operons, we next constructed promoter-*luxABCDE* fusions that were inserted into the *sacA* locus of the *B. subtilis* wild type strain W168, resulting in strains P_*liaI*_ (TMB3822), P_*yhbI*_ (TMB5779), P_*mdtR*_ (TMB5780) and P_*lnrL*468_ (TMB4220) (Table 1). We tested their activation but failed to observe amphotericin B-dependent induction for P_*liaI*_, P_*yhbI*_ and P_*mdtR*_ (data not shown). In addition, single mutants lacking the *liaIH* operon, the *yhbIJ-yhcABC* operon, the *mdtRP* operon and double mutants of *liaIH* combined with *lnrLMN* (strains TMB1151, TMB5771, TMB5772 and TMB4241, Table 1), showed no phenotype when challenged with amphotericin B (data not shown). However, we observed a strong, dose-dependent induction of P_*lnrL*468_ in response to amphotericin B (Figure 3F and Supplementary Figure S2). The global response of *B. subtilis* to amphotericin B treatment therefore does not mount a specific resistance and mainly affects the expression of the *lnrLMN* operon alone, which showed an induction of over 500-fold (Figure 2 and Table 3).

Furthermore, own unpublished results comparing the amphotericin B induced transcriptome with that of nine other non-polyene antibiotics indicates that the transcriptome pattern for amphotericin B is indeed unique. With the exception of *liaIH* operon, which is more strongly induced by bacitracin and vancomycin, the induction of the eleven remaining genes, and particularly the induction of the *LnrLMN* operon, is specifically triggered by amphotericin B exposure (Zhang et al. personal communication). This highly specific cellular response of *B. subtilis* upon amphotericin B exposure without having any effect on growth and the strong induction of the *lnr* operon motivated us to further characterize this system to develop a whole-cell biosensor.

### Induction Spectrum of the *lnr* System in *B. subtilis*

The *lnrJKLMN* locus of *B. subtilis* W168 (*BSU08290-BSU08330*, formerly *yfiJKLMN*) encodes a TCS, LnrJK, and an ABC transporter, LnrLMN (Figure 3A; Yamamoto et al., 1996). TCSs consist of a membrane-anchored histidine kinase (HK) and a cytoplasmic response regulator (RR). Upon stimulus perception, the HK autophosphorylates at a histidine residue. Subsequently, the high energy phosphate group is specifically transferred to an aspartyl residue in the cognate RR leading to its activation. As a consequence, the activated RRs usually mediate the cellular output, often by acting as transcriptional activators/repressors (Stock et al., 2000). In *B. subtilis* NCIB3610, the TCS LnrJK regulates the expression of the LnrLMN transporter, which confers resistance to linearmycins: long linear polyene antibiotics (Figure 1) with antifungal and antibacterial activity, isolated from *Streptomyces* sp. no. 30 (Sakuda et al., 1996; Stubbendieck and Straight, 2015). A reporter strain, P_*yfiLMN*_-*lacZ* was activated by linearmycins and ECO-02301 and weakly induced by the non-lytic amphotericin B and nystatin, while it was not induced by the lytic lipopeptide daptomycin (Stubbendieck and Straight, 2017). The authors concluded that the signaling leading to promoter activation was specific for linearmycins, and hence renamed the system to *lnrJKLMN* for linearmycin sensing and response (Stubbendieck and Straight, 2015, 2017).

We next studied the response of the P_*lnrL*468_-lux reporter strain in liquid medium to the commercially available cyclic polyenes filipin III, natamycin, and nystatin (Figure 1). Filipin III and natamycin did not activate P_*lnrL*468_ (Figures 3G,H), but we observed a weak induction of P_*lnrL*468_ by nystatin (Figure 3I), a compound structurally related to amphotericin B (Figure 1). Our results supported the previously reported weak induction of the *lnrL* promoter after spotting nystatin and amphotericin B on top of *B. subtilis* P_*yfiLMN*_-*lacZ* colonies (Stubbendieck and Straight, 2017). We next aimed at determining the minimal polyene-responsive *lnrL* promoter, in order to develop the P_*lnrL*_ promoter into a whole-cell biosensor.

### Optimization of the *lnrL* Promoter to Develop a Whole-Cell Biosensor

**FIGURE 4:**
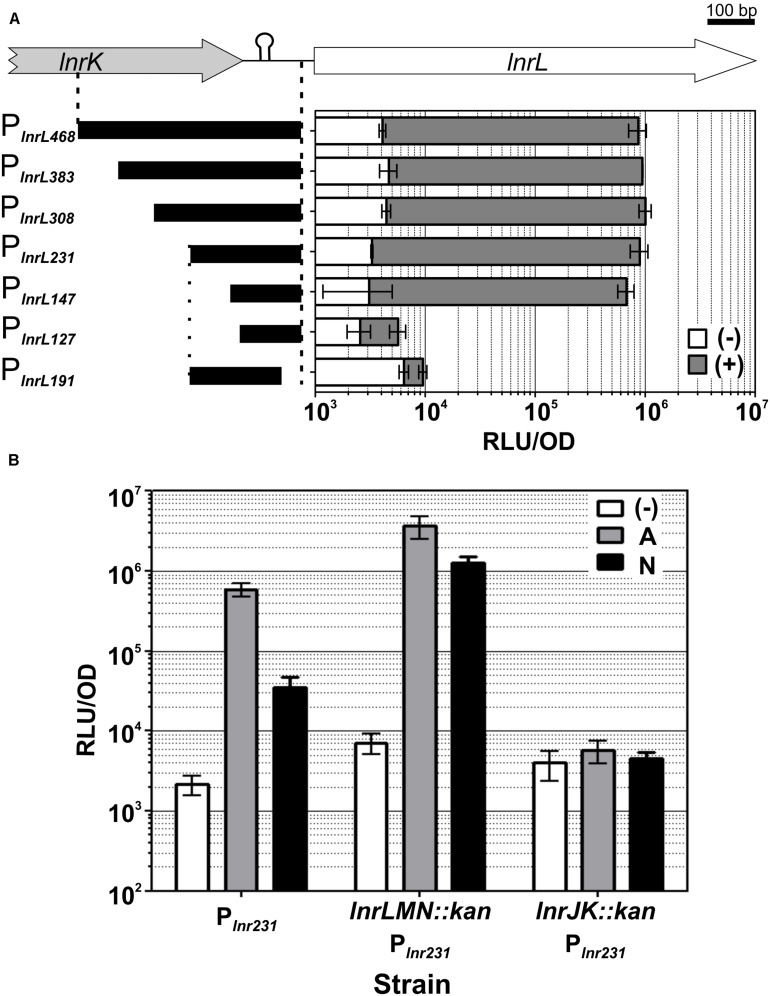
LnrJKLMN system characterization. **(A)** Identification of the minimal *lnrL* promoter fragment. The genetic region containing the P_*lnrL*_ promoter, the promoter truncations constructs and their functional analysis are presented. The genes *lnrK* (block gray arrow) and *lnrL* (block white arrow) are depicted; the length of the initial P_*lnrL*468_ promoter is indicated by vertical dashed lines. The vertical dotted line indicates the beginning of the 3’ promoter truncation. Rho-independent transcriptional terminator between *lnrK* and *lnrL* is indicated by a hairpin. Schematics of P_*lnrL*_ promoter truncations are drawn as black bars on the left side. Genes and promoter fragments drawn to scale. Promoter activities one hour after induction with 4 μg ml^– 1^ of amphotericin B (gray bars) relative to the activities of the uninduced promoters at the same time point (white bars), are presented in the right graph as relative luminescence units by OD_600_. **(B)** Mechanism of sensing. The activity of P_*lnrL*231_ in response to amphotericin B and nystatin was determined in a wild type background (strain TMB4223), in a mutant eliminated in the *lnrLMN* transporter (strain TMB5423) and in a mutant eliminated in the *lnrJK* TCS (strain TMB5422). The promoter activity at one-hour post induction with amphotericin B 4 μg ml^– 1^ (gray) and nystatin 167 μg ml^– 1^ (black) is presented relative to the uninduced state (white), as relative luminescence units by OD_600_. The chosen concentrations of the compounds correspond to the maximum promoter induction, as indicated by the data shown in Fig. 5. The experiments were performed in quadruplicate with two independent clones; means and standard deviations are depicted.

Toward this goal, we progressively truncated the *lnrL* promoter fragment, starting at the 5′-position from P_*lnrL*468_ to a final promoter length of 127 base pairs, and generated corresponding transcriptional *luxABCDE* reporter fusions (Table 1). All of the promoter constructs ended at the identical 3′-end as the previous fragment, that is, −16 base pairs relative to the GTG start codon of *lnrL* (Figure 4A). In case of the 3′-truncation, the promoter extended from the 5′-end of P_*lnrL*231_ until - 57 base pairs relative to the *lnrL* start codon (Figure 4A). The promoter fusions were integrated into the *sacA* locus in *B. subtilis* W168 wild type and the promoter activity of the resulting reporter strains (Table 1) was determined by a quantitative luminescence assay after induction with 4 μg ml^–1^ of amphotericin B, since this concentration was sufficient to generate full P_*lnrL*468_ induction (Supplementary Figure S2C).

The progressively 5′-end truncated *lnrL* promoters P_*lnrL*383_, P_*lnrL*308_ and P_*lnrL*231_ showed activities comparable to P_*lnrL*468_ after amphotericin B addition (Figure 4A gray bars). While P_*lnrL*147_ showed an almost negligible decreased promoter activity, a further truncation of 20 nucleotides in P_*lnrL*127_ led to a complete loss of inducible promoter activity. The 3′-truncation P_*lnrL*191_ starting at the 5′ end of P_*lnrL*231_ until - 57 base pairs relative to the *lnrL* start codon also led to a complete loss of promoter activity after amphotericin B addition (Figure 4A). We therefore proceeded our characterization with P_*lnrL*231_, thereby ensuring the coverage/inclusion of all potential regulatory elements of the promoter and optimal promoter activity (Figure 4A).

### Characterization of the LnrJKLMN System Regulation: Boosting Output to Background Ratio

The LnrLMN ABC transporter was reported to confer resistance to linearmycins but not to be required for signaling the presence of the linearmycins to the TCS LnrJK (Stubbendieck and Straight, 2015, 2017). Since P_*lnrL*_ is strongly induced by amphotericin B and weakly by nystatin (Figure 3), we next investigated if the LnrJKLMN system mediates amphotericin B and/or nystatin resistance. For that goal, two mutant strains (TMB4238 and TMB4237, Table 1) were constructed that eliminated either *lnrJK*, encoding the TCS, or *lnrLMN*, encoding the ABC transporter. These strains were then subjected to sensitivity assays in liquid medium with increasing serial dilutions of amphotericin B (from 4 to 64 μg ml^–1^) and nystatin (from 42 to 333 μg ml^–1^). No difference was observed for both strains compared to the wild type strain (data not shown), indicating that the LnrJKLMN system does not mediate any resistance to these two polyenes.

While LnrLMN is not involved in mediating amphotericin B or nystatin resistance, we still wondered if this ABC transporter might nevertheless be involved in sensing these polyene compounds. This idea was inspired by the analogous Bce-like modules involved in antimicrobial peptide resistance in Firmicutes (named after the prototypical BceRSAB system of *B. subtilis*), which consist of an ABC transporter (BceAB) that senses and signals the presence of the antimicrobial peptide bacitracin to its associated TCS (BceRS). BceR, in turn, strongly induces *bceAB* expression to ultimately mediate high-level bacitracin resistance (Ohki et al., 2003; Rietkötter et al., 2008; Fritz et al., 2015). Consequently, we next investigated if the transporter LnrLMN is required for amphotericin B and nystatin perception and signaling to the TCS LnrJK. We therefore deleted *lnrJK* and *lnrLMN* in the P_*lnrL*231_-*lux* reporter strain, resulting in strains TMB5422 and TMB5423, respectively. These strains were subjected to induction assays with amphotericin B (4 μg ml^–1^) and nystatin (167 μg ml^–1^). While the absence of the TCS LnrJK completely abolished promoter induction, as expected, the absence of the ABC transporter LnrLMN led to a higher P_*lnrL*231_ activity compared to the wild type (Figure 4B): we observed a six-fold increased induction in response to amphotericin B and a 36-fold increased induction in response to nystatin in the *lnrLMN* mutant strain, relative to the corresponding wild type strain. This result demonstrates that the LnrLMN transporter is not involved in polyene sensing, in line with a postulated repressory function of LnrLMN on LnrJK-dependent signaling (Stubbendieck and Straight, 2017), the molecular nature of which remains to be uncovered.

### Development and Validation of a Whole-Cell Biosensor Based on the *lnrL* Promoter

Taken together, our results show that (i) the *lnrLMN* operon is the only regulatory target of LnrK and (ii) that LnrLMN exerts an inhibitory effect on LnrJK signaling. For the final whole-cell biosensor strain, an *lnrLMN* clean deletion (strain TMB5578, Table 1) was therefore combined with the P_*lnrL*231_-*lux* construct into strain TMB5600 (Δ*lnrLMN P_*lnrL*231_-lux*, Table 1) to (i) decrease the metabolic burden of *lnrLMN* expression upon P_*lnrL*_ induction, and (ii) optimize biosensor sensitivity and dynamics. Its performance as a polyene whole-cell biosensor was subsequently comprehensively analyzed.

**FIGURE 5:**
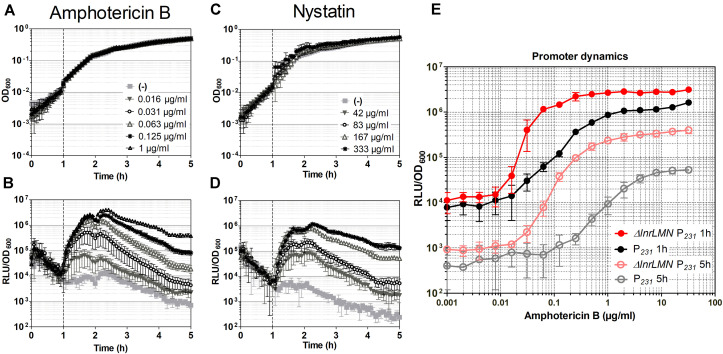
Characterization of the Δ*lnrLMN* P_*lnrL*231_-*lux* (TMB5600) whole-cell biosensor. **(A–D)** Induction of the Δ*lnrLMN* P_*lnrL*231_ (TMB5600) by amphotericin B and nystatin in liquid media. The effect of antibiotic exposure on growth is indicated as OD_600_ **(A)** and **(C)**; the promoter induction is presented as relative luminescence units by OD_600_ **(B)** and **(D)**. The time of antibiotic addition is indicated with a vertical black dashed line. The antibiotic concentrations used are indicated in the upper panels. **(E)** Effect of deletion of *lnrLMN* genes on P_*lnrL*231_ promoter dynamics. The amphotericin B-concentration dependent promoter induction when the LnrLMN transporter is present (P_231_, strain TMB4223) or absent (Δ*lnrLMN* P_231_, strain TMB5600) on the cell is presented. The values correspond to promoter activities one and five hours after induction with serial dilutions of amphotericin B. The symbols color code is indicated in the graph. The experiments were performed at least in triplicate with two independent clones. Means and standard deviations are depicted.

**FIGURE 6:**
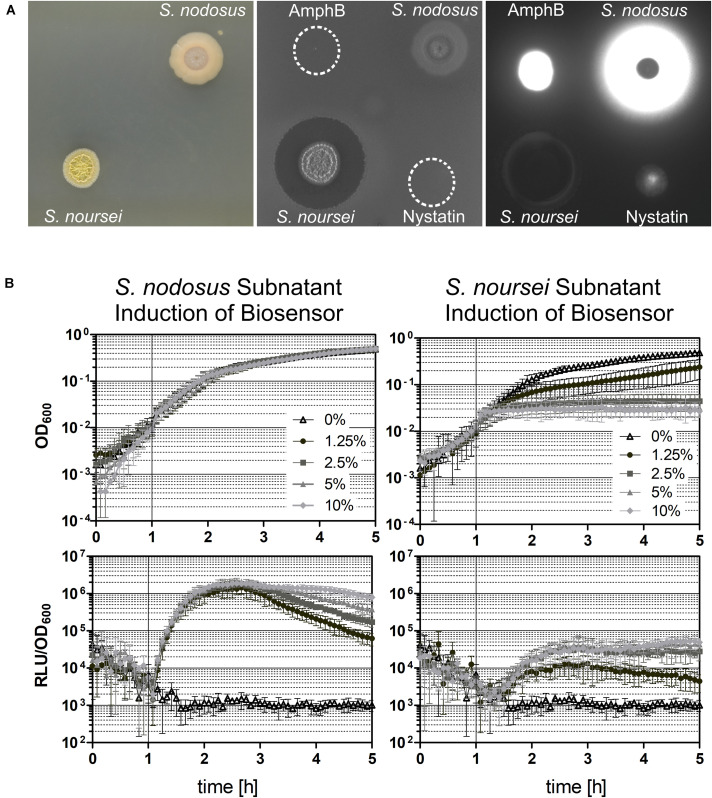
Induction of the Δ*lnrLMN* P_*lnrL*231_-*lux* (TMB5600) biosensor with *Streptomyces* spp. subnatants. The biosensor was tested for its robustness by analyzing its ability to detect polyenes in a complex mixture of different produced compounds by the two *Streptomyces* species *S. nodosus* and *S. noursei*, which are known producers of amphotericin B and nystatin, respectively. **(A)** Spot-on-lawn in solid medium. Both *Streptomyces* spp. strains were grown on solid medium for 4 days at 28°C (left picture), before they were overlaid with the biosensor strain (middle picture) and the produced luminescence signal was detected (right picture). In addition, 20 μl of amphotericin B (AmphB) and nystatin stock solutions were spotted on top of the overlay as controls. **(B)** Induction in liquid medium. The Δ*lnrLMN* P_*lnrL*231_-*lux* biosensor was also analyzed in liquid conditions by inducing exponentially growing cells with different concentrations of the subnatants produced by the two *Streptomyces* species. The top panels illustrate the growth of the biosensor strain (indicated as OD_600_) and the bottom panels show the induction with the subnatants produced by *S. nodosus* (left) and *S. noursei* (right) (as relative luminescence units by OD_600_). The graphs depict the behavior of the biosensor over the course of five hours as means and standard deviations of three independent experiments.

We characterized the robustness of the Δ*lnrLMN P*_*lnrL*231_ whole-cell biosensor by checking its induction in liquid and in plate-based assays. For the induction assays in liquid, the cells were challenged with amphotericin B and nystatin in exponential phase, and growth and luminescence readouts were monitored over time. The activation on solid medium was determined by spot-on-lawn assays: a lawn of the *B. subtilis* biosensor in soft agar was challenged with 20 μl of stock solutions of the macrolide polyenes and the luminescence output was determined after 24 h. In liquid conditions, the biosensor showed an over 100-fold induction in the presence of both compounds (Figures 5B,D). The dynamic range of *P*_*lnrL*231_ in a Δ*lnrLMN* background is over 10-fold higher than that observed in the wild type and particularly pronounced for nystatin (compare Figure 3I with Figure 5D). The final biosensor also demonstrated its sensitivity in solid media, where Δ*lnrLMN P*_*lnrL*231_ produced a significantly stronger luminescence output for amphotericin B and a weaker but detectable luminescence output for nystatin (Figure 6A).

We next determined the dose-response kinetics by challenging our biosensor (TMB5600) – and the isogenic wild type predecessor strain (TMB4223) – with serial dilutions (from 0.001 to 32 μg ml^–1^) of amphotericin B. Our results highlight that removing *lnrLMN* improves the promoter performance with regard to both sensitivity and dynamic range (Figure 5E). P_*lnrL*231_ in the wild type background has a gradual increase in promoter induction (Figure 5E, black line), highlighting its potential to resolve a more linear dose-response to the inducing agent. However, the Δ*lnrLMN* P_*lnrL*231_ reporter strain has a sigmoidal response with a sharper off-on activation resulting in higher sensitivity (Figure 5E, red line). For example, at one hour post-induction an amphotericin B concentration of 1 μg ml^–1^ was required to detect a reporter activity of 10^6^ RLU/OD_600_ with P_*lnrL*231_ in a wild type background, however, an almost 16-fold lower concentration of 0.063 μg ml^–1^ was enough to induce the same luminescence output with strain Δ*lnrLMN* P_*lnrL*231_. The same difference in dynamic range is maintained at 5 h post induction (Figure 5E), but there is a significant decrease in background signal, relative to the background signal at 1-h post induction, for both strains.

Overall, our reporter strains therefore show a low background signal, and a highly dynamic, dose-dependent response that covers more than two orders of magnitude in range, which makes both TMB4223 (wild type) and TMB5600 (Δ*lnrLMN*) superbly performing whole-cell biosensors (Figures 3, 5). While this quantitative output with pure compounds is impressive, whole-cell biosensors are usually applied in a more qualitative setting to screen either raw culture extracts or potential producing strains directly. We therefore wanted to also demonstrate the potential of our biosensor by screening the producers of amphotericin B and nystatin, *S. nodosus* ATCC14899 and *S. noursei* (Caffrey et al., 2001; Fjaervik and Zotchev, 2005), for their ability to induce the Δ*lnrLMN* P_*lnrL*231_ biosensor. The results (Figure 6) show no zone of inhibition – in agreement with the preference for macrolide polyenes for fungal membranes – but a bright luminescence halo around the *S. nodosus* colony, comparable in intensity to the amphotericin B halo (Figure 6A). On the other hand, *S. noursei* created a zone of inhibition for *B. subtilis*, while there is only a very slight luminescence halo detectable at the very rim of the inhibition zone (Figure 6A). This observation fits the weak luminescence signal observed for the pure compound, nystatin (Figure 6A). These results could be corroborated in liquid: *S. nodosus* and *S. noursei* were grown on top of liquid medium and the harvested subnatant was used to induce exponentially growing *B. subtilis* TMB5600 biosensor cells (Figure 6B). The *S. nodosus* subnatant induced the biosensor equally strong as amphotericin B (Figure 5) and had no effect on growth, while the *S. noursei* subnatant heavily impaired growth and therefore no luminescence signal could be detected. Since no interference of pure nystatin with the growth of *B. subtilis* was observed (Figures 5C, 6A), the observed growth impairment in the presence of *S. noursei* is most likely due to the production of another antibacterial compound by this *Streptomyces* species (Abou-Zeid and El-Sherbini, 1974; Wu et al., 2009).

### Investigation of the Whole-Cell Biosensor Specificity

The Δ*lnrLMN* P_*lnrL*231_ biosensor is activated by amphotericin B and nystatin (Figure 5) and it was described that a reporter strain, P_*yfiLMN*_-*lacZ*, in *B. subtilis* NCIB3610, was activated by linearmycins and ECO-02301 (Stubbendieck and Straight, 2017). Interestingly, natamycin and filipin III fail to induce the most sensitive biosensor TMB5600 in liquid and in solid (data not shown), suggesting that the absence of promoter induction is due to a lack of recognition by the LnrJ histidine kinase rather than a poor sensitivity of the detection/reporter system. Furthermore, a control study testing the response of Δ*lnrLMN* P_*lnrL*231_ biosensor to a collection of 13 non-polyene antibiotics (Supplementary Figure S3) of different chemical natures (Supplementary Figure S4), also showed no induction of the most sensitive TMB5600 biosensor, further supporting the specific recognition of some polyene compounds by the LnrJ histidine kinase.

Linearmycins and ECO-02301 are linear polyenes (Figure 1), while amphotericin B and nystatin are macrolide polyenes (Figure 1), all of which are produced by *Streptomyces* species (Sakuda et al., 1996; Caffrey et al., 2001; Fjaervik and Zotchev, 2005; McAlpine et al., 2005). According to the polyene classification (Hamilton-Miller, 1973; Madden et al., 2014), linearmycins and ECO-02301 are both pentaenes (Sakuda et al., 1996; McAlpine et al., 2005); amphotericin B is an heptaene and the structurally related nystatin is a tetraene that has been called a “degenerated heptaene” since it has one double bond reduced (a saturated bond) that separates a diene and a tetraene region in the chromophore (Kotler-Brajtburg et al., 1979; Figure 1). Linearmycins and ECO-02301 lyse *B. subtilis* cells, whereas amphotericin B and nystatin do not (Stubbendieck and Straight, 2017). The activity of linearmycins on *B. subtilis* cells is due to cytoplasmic membrane depolarization (Stubbendieck et al., 2018). Our experimental results [following established protocols described in te Winkel et al. (2016)] showed that amphotericin B at a concentration of 10 μg ml^–1^ and nystatin at 83 μg ml^–1^ do not depolarize the *B. subtilis* cytoplasmic membrane (data not shown). These results rule out that membrane depolarization is the stimulus for the activation of LnrJK TCS.

Interestingly, in 1979 Kotler-Brajtburg et al. classified fourteen polyene antibiotics and six semisynthetic derivatives in two groups with different mechanism of action on mouse erythrocytes and *Saccharomyces cerevisiae* (Kotler-Brajtburg et al., 1979). For Group I antibiotics, hemolysis or cell death and K^+^ leakage were caused at the same concentrations of added polyene, while for Group II antibiotics, K^+^ ion leakage was caused at low polyene concentrations and hemolysis or cell death at high polyene concentrations. Within this classification by differential mode of action on eukaryotic cells, natamycin and filipin belong to Group I whereas amphotericin B and nystatin belong to Group II. This classification was later supported by the work of Akiyama et al. concerning the concentration of polyenes required to inhibit the colony formation of Chinese hamster V79 or *Saccharomyces cerevisiae* cells (Akiyama et al., 1980). Considering our results, showing no induction of P_*lnrL*_ by natamycin and filipin III (Group I), and the induction by amphotericin B and nystatin (Group II) (Figure 3), and previous studies showing the activation of the promoter by the linear polyenes linearmycins and ECO-02301 (Stubbendieck and Straight, 2017), we propose that *B. subtilis* Δ*lnrLMN* P_*lnrL*231_ is a biosensor specific for such large linearmycin-like and amphotericin-like (Group II) polyenes. The full spectrum of LnrJ-sensed polyenes, as well as the potential similarities these linear and cyclic polyenes share, for example regarding mechanism of action over eukaryotic cells, will be the focus of future investigations.

## Conclusion

Our results show that challenging *B. subtilis* with bioactive molecules that are not antibacterial but might occur in the same soil microbiome habitat, could indeed be a very promising strategy to identify new biosensor candidates for expanding the toolbox to screen for novel antibiotics, including antifungal polyenes. In this work, we investigated *B. subtilis* perception *of* and response *to* the bioactive, antifungal secondary metabolite amphotericin B, which is produced by *Streptomyces* spp. While this compound does not inhibit *B. subtilis*, it strongly and specifically induces the *lnrLMN* operon in a LnrJK-dependent manner. The LnrLMN ABC transporter has a physiological relevance by mediating linearmycin resistance in *B. subtilis* (Stubbendieck and Straight, 2015). However, besides sensing damage-inducing linearmycins and ECO-02301 (Stubbendieck and Straight, 2017), LnrJK TCS has a wider (but specific) sensitivity for non-damaging polyenes such as amphotericin B and nystatin. Using *B. subtilis* molecular tools, we created Δ*lnrLMN* P_*lnrL*231_ (TMB5600), a sensitive reporter strain based on the P_*lnrL*_ target promoter of the antibiotic-responsive LnrJK two-component signaling system, with a direct application in identification and discovery of antifungal-drugs. The biosensor dynamics was characterized, and it was subsequently applied to identify known *Streptomyces* spp. producers of natural polyenes. We have shown that the P_*lnrL*231_ promoter is switched off in the absence of its inducers and responds in a dynamic, dose-dependent manner to increasing concentrations of them, providing an over 100-fold induction over background activity. The new biosensor is very sensitive and has proven to be robust working under different experimental conditions and responding to pure compounds as well as inducing compounds in complex samples such as *Streptomyces* subnatants. The Δ*lnrLMN* P_*lnrL*231_ (TMB5600) biosensor is ready to serve the discovery of novel polyenes with potential application in human medicine.

## Data Availability Statement

The raw data supporting the conclusions of this article will be made available by the authors, without undue reservation, to any qualified researcher. The raw and processed RNA sequencing data obtained in this study has been deposited at the NCBIs Gene Expression Omnibus (Edgar et al., 2002) and is accessible via the GEO accession number GSE148903.

## Author Contributions

AR-G, FD, PP, and TM conceived the study. AR-G wrote the manuscript with the help of FD and PP, under the supervision of TM. AR-G, FD, and PP planned the experiments. AR-G, FD, PP, and MD carried out all the experiments with *B. subtilis* and analyzed the data; the experiments with the *Streptomyces* strains were carried out by FD. All authors read the article and agreed on the final manuscript.

## Conflict of Interest

The authors declare that the research was conducted in the absence of any commercial or financial relationships that could be construed as a potential conflict of interest.

## Supplementary Material

FIGURE S1 Control assays with the P_*lnrL*468_ promoter (strain TMB4220).

FIGURE S2 Amphotericin B dose response with P_*lnrL*468_ promoter (strain TMB4220).

FIGURE S3 Control study testing the response of Δ*lnrLMN* P_*lnrL231*_ biosensor to a collection of 13 non-polyene antibiotics (strain TMB5600).

FIGURE S4 Molecular structures of the non-polyene antibiotics used in the control study presented in Supplementary Figure S3.

TABLE S1 Oligonucleotides used in this study.
