# Supplementary material for: Amphotericin B Specifically Induces the Two-Component System LnrJK: Development of a Novel Whole-Cell Biosensor for the Detection of Amphotericin-Like Polyenes
Source: Front Microbiol. 2020 Aug 21;11:2022. doi: 10.3389/fmicb.2020.02022 (PMC7472640; doi:10.3389/fmicb.2020.02022)
Supplement: FIGURE S1 — Control assays with the PlnrL468 promoter (strain TMB4220). [file Data_Sheet_1.PDF]

Supplementary Material

1 Supplemental Table 1. Oligonucleotides used in this study

Name and purpose		Description (sequence)[a]	Use
**Promoter fusions**			
TM5098	PlnrLMN -XbaI.fw	AAAAtctagaCAGATACGATTGTAAAAGCAGTG	Promoter truncations
TM5694	PlnrLMN.Fw	TTTT**gaattccgcggccgcttctaga**CATATGACGTTTTGCATATACG	
TM5695	PlnrLMN.Rv	TTTT**ctgcagcggccgctactagta**TTTTGATCCATTGCCATTGTAG	
TM5787	PlnrLMN383.Fw	TTTT**gaattccgcggccgcttctaga**ATGGAAGCGTGAAAAACAGC	
TM5788	PlnrLMN308.Fw	TTTT**gaattccgcggccgcttctaga**CGAAATCGAAGTTCTGGCTG	
TM5789	PlnrLMN231.Fw	TTTT**gaattccgcggccgcttctaga**GCACAGTCAAAAACCACGTT	
TM5855	PlnrLMN147.Fw	TTTT**gaattccgcggccgcttctaga**ATGGTGTATCTGTCTTTTGAC	
TM6014	PlnrLMN191.Rv	TTTT**ctgcagcggccgctactagta**GCATGTAGCGGCAGATAGAAC	
TM6334	PyhbI-fwd	AGCT**gaattcgcggccgcttctaga**GATTTAAGTACTATATTTTAAAA CAGAAATCTG	RNA sequencing result confirmation
TM6335	PyhbI-rev	AGCT**ctgcagcggccgctactagta**AAGATAAAATATTAATAAATGA AAGAAGATTTG	
TM6336	PmdtR-fwd	AGCT**gaattcgcggccgcttctaga**GTCCAATATGGATGGTATTCTG	
TM6337	PmdtR-rev1	AGCT**ctgcagcggccgctactagta**TTATTAAATATAAATAAGCGTAA ATGAAAAGTC	
**Check primers for pBS3C*lux***			
TM2262	pAH328checkfwd	GAGCGTAGCGAAAAATCC	sequencing
TM2263	pAH328checkrev	GAAATGATGCTCCAGTAACC	
TM2505	pAH328 sacA front check fwd	CTGATTGGCATGGCGATTGC	integration of up fragment into genome
TM2506	pAH328 sacA front check rev	ACAGCTCCAGATCCTCTACG	
TM5955	pBS3Clux back check rev	GCAGCCTTTTCCAAACATTCCG	Integration of down fragment into genome
TM5956	pBS3Clux back check fwd	GATAGTTGATATCCAGCAGGATC	
TM2507	pAH328 sacA back check fwd	GTCGCTACCATTACCAGTTG	
TM2508	pAH328 sacA back check rev	TCCAAACATTCCGGTGTTATC	
**Gene knock out via Long-Flanking-Homology**			
TM0137	kan-fwd	CAGCGAACCATTTGAGGTGATAGG	kanamycin cassette
TM0138	kan-rev	CGATACAAATTCCTCGTAGGCGCTCGG	
TM0139	mls-fwd	CAGCGAACCATTTGAGGTGATAGGGATCCTTTAACTCTGG CAACCCTC	mls cassettes
TM0140	mls-rev	CGATACAAATTCCTCGTAGGCGCTCGGGCCGACTGCGCAA AAGACATAATCG	
TM0056	kan-check-fwd	CATCCGCAACTGTCCATACTCTG	Check allelic replacement
TM0147	kan-check-rev	CTGCCTCCTCATCCTCTTCATCC	
TM0148	mls-check rev	GTTTTGGTCGTAGAGCACACGG	

TM0057	mls-checkfwd	CCTTAAAACATGCAGGAATTGACG	
TM5794	LFH-LMN-up-fwd	ACGGCAAAGGCAATCCTGAG	Knock-out of *lnrLMN* operon
TM5795	LFH-LMN-up-rev	CCTATCACCTCAAATGGTTCGCTGTCCGTATGCCTTTTTGATGTTTTCTGC	
TM5796	LFH-LMN-do-fwd	CGAGCGCCTACGAGGAATTTGTATCGTGCTTGCATTTGCTGCGATC	
TM5797	LFH-LMN-do-rev	TGACTCAGCTTTTGCGCCAGAC	
TM5802	LFH-JK-up-fwd	ATGAGATTTGTAAGGCGGCG	Knock-out of *lnrJK* operon
TM5803	LFH-JK-up-rev	CCTATCACCTCAAATGGTTCGCTGTGGACAGATACATTAAACAGCTCACC	
TM5804	LFH-JK-do-fwd	CGAGCGCCTACGAGGAATTTGTATCGACCCAAGCCGCCATTTACTC	
TM5805	LFH-JK-do-rev	TCCTGCACCTGAAGAGACAG	
TM6339	yhb-yhc_up-fwd	AGGCGAGGATTATTATGAAGCG	Knock-out of *yhb-yhc* operon
TM6340	yhb-yhc_up-rev	CCTATCACCTCAAATGGTTCGCTGAGCACGTTCTGATTCAGTCAAC	
TM6341	yhb-yhc_do-fwd	CGAGCGCCTACGAGGAATTTGTATCGATGAAAAATAAGAAAAAGTAATGAAAAAGGC	
TM6342	yhb-yhc_do-rev	ATTTCTCTTACTGAAGGAAGCTTG	
TM6343	mdtRP_up-fwd	ACAAACCGGATCTGTTTAGC	Knock-out of *mdtRP* operon
TM6344	mdtRP_up-rev	CCTATCACCTCAAATGGTTCGCTGTAACTGATCCGCACTCTTCATATTC	
TM6345	mdtRP_do-fwd	CGAGCGCCTACGAGGAATTTGTATCGGTTGAATCACCTTCCCATTAATAGAG	
TM6346	mdtR_do-rev	AGTGGAAATGATTCAAAAAGCTG	
**Clean deletion**			
TM6094	deltaLMN-fw	ATGCGAATTCGCACTTGGTTTTGGTCTCTTG	Amplification of joined *lnrLMN* fragment
TM6095	deltaLMN-rev	ATGCGGATCCGCGCCAGACGGCGGAGCG	
TM253	pMAD-checkrev	GTTACGTTACACATTAACTAGAC	check for integration of pMAD-*lnrLMN*
TM6026	ch.integr.up-rev-LMN	ATCATCGAGATGGTTGTGG	
TM254	pMAD-checkfwd	CTGATGGTCGTCATCTACCTGCC	
TM6027	ch.integr.down-fwd-LMN	TGCGATCACTTTCGCAGCGG	
TM6028	chdl-LMN-fwd	AGCCTTGTCATTAAGGATGAC	Check for *lnrLMN* deletion in genome and sequencing
TM6029	chdl-LMN-rev	CCATGAACAAGAACGACAGG	
TM5694	PlnrLMN.Fw	TTTTGAATTCCGCGGCCGCTTCTAGACATATGACGTTTTGCATATACG	

[a] Sequences are given in the 5′→ 3′direction. Restriction sites are shown in lowercase. Biobrick (Radeck, Kraft et al. 2013) overhangs are shown in bold lowercase. Sequences underlined are inverse and complementary to the 5’ (up-reverse) and 3’ (do-forward) ends of the kanamycin and mls cassettes.

Radeck, J., K. Kraft, et al. (2013). "The Bacillus BioBrick Box: generation and evaluation of essential genetic building blocks for standardized work with Bacillus subtilis." Journal of Biological Engineering **7**(1): 29.

2 Supplementary Figures

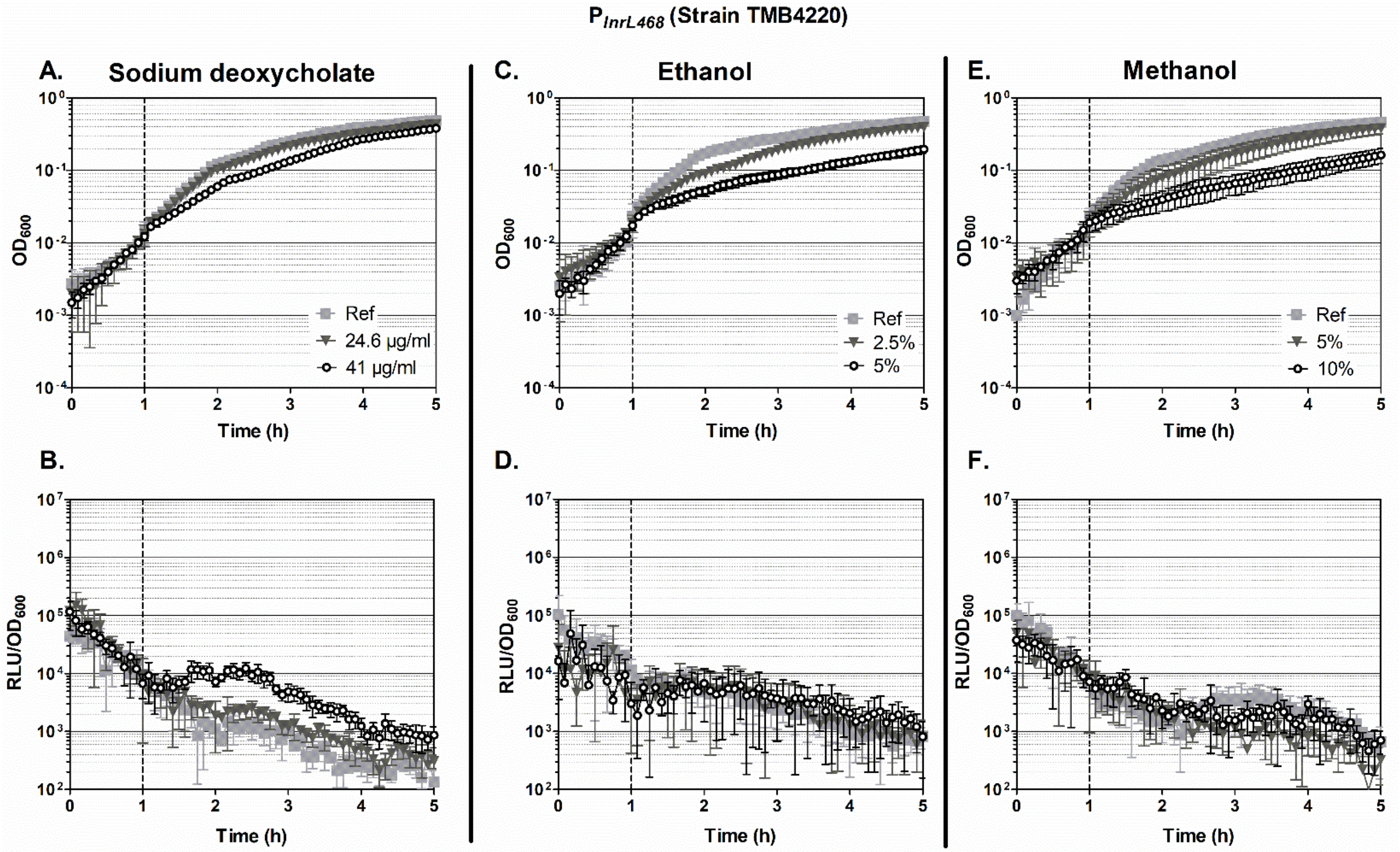

Supplementary Figure 1. Control assays with the $P_{lnrL468}$ promoter (strain TMB4220). The effects of the solvents sodium deoxycholate, ethanol and methanol on *B. subtilis* growth and on the $P_{lnrL468}$ activation were determined by means of sensitivity (indicated as OD_{600}) and induction assays (indicated as relative luminescence units by OD_{600}). The time of solvent addition is indicated with a vertical dashed line. The concentrations of the solvents used are indicated in the upper panel graphs. The experiments were performed at least in triplicate with two independent clones. Means and standard deviations are depicted.

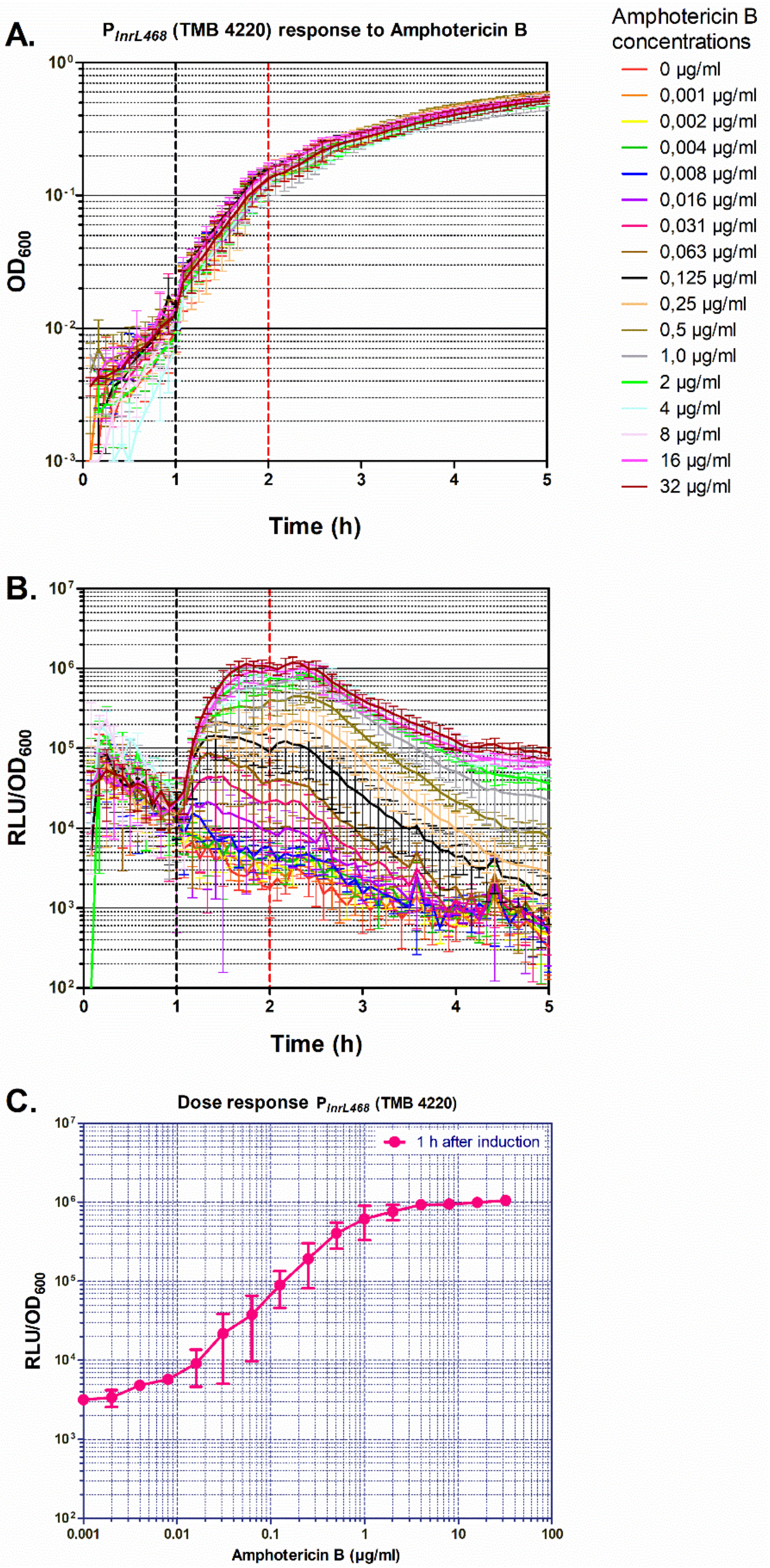

Supplementary Figure 2. Amphotericin B dose response with $P_{lnrL468}$ promoter (strain TMB4220). **A)** Effect of exposition to serial dilutions (from 0.001 to 32 µg/ml) of amphotericin B on growth of $P_{lnrL468}$ reporter strain (TMB4220) indicated as OD_{600}. **B)** $P_{lnrL468}$ induction (as relative luminescence units by OD_{600}) resulting from exposition to the same concentrations of amphotericin B as in (**A**). **C**) Amphotericin B dose-response curve of the $P_{lnrL468}$ promoter at a fixed time (1 hour after induction). In **(A)** and **(B)**, the time of antibiotic addition is indicated with a vertical black dashed line; the selected time (one hour after induction) for the dose response presented in **(C)** is indicated with a vertical red dashed line. The experiments were performed in triplicate with two independent clones. Means and standard deviations are depicted.

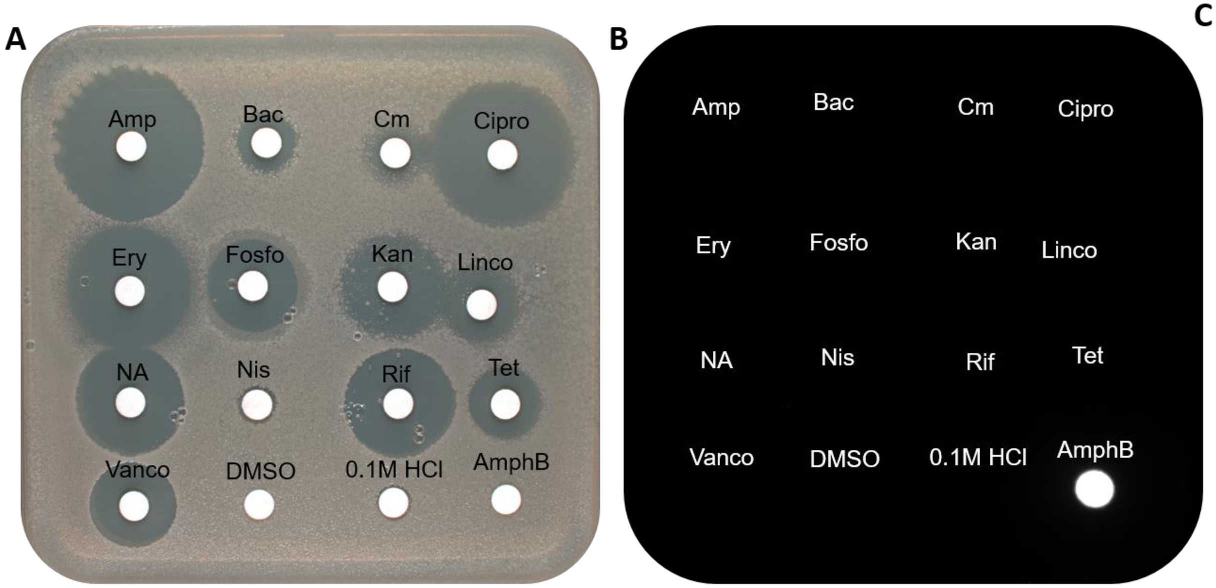

C

Compound	Concentration	Solvent
Ampicillin **(Amp)**	10 mg ml^{-1}	dH_2O
Bacitracin **(Bac)**	20 mg ml^{-1}	dH_2O
Chloramphenicol **(Cm)**	1 mg ml^{-1}	70% EtOH
Ciprofloxacin **(Cipro)**	0.16 mg ml^{-1}	HCl
Erythromycin **(Ery)**	0.5 mg ml^{-1}	70% EtOH
Fosfomycin **(Fosfo)**	10 mg ml^{-1}	dH_2O
Kanamycin **(Kan)**	1 mg ml^{-1}	dH_2O
Lincomycin **(Linco)**	2.5 mg ml^{-1}	dH_2O
Nalidixic acid **(NA)**	2.5 mg ml^{-1}	dH_2O
Nisin **(Nis)**	1 mg ml^{-1}	dH_2O
Rifampicin **(Rif)**	1 mg ml^{-1}	DMSO
Tetracycline **(Tet)**	1.25 mg ml^{-1}	dH_2O
Vancomycin **(Vanco)**	1 mg ml^{-1}	dH_2O
Amphotericin B **(AmphB)**	250 mg ml^{-1}	dH_2O

Supplementary Figure 3. **Control study testing the response of Δ*lnrLMN* P$_{lnrL231}$ biosensor to a collection of 13 non-polyene antibiotics (strain TMB5600).** The spot-on-lawn assay was performed as described in the Materials and Methods section of the main text, with the modifications described next. The day cultures of the biosensor strain TMB5600 were incubated at 37 °C (220 rpm) until an OD_{600} of around 0.4-0.7 was reached. Then, the cell suspensions were diluted to an OD_{600} of 0.01 in 15 ml of LB-soft (0.75%) agar, homogenized by shortly vortexing, and poured on top of the LB agar plates. After a drying period of at least 10 minutes, the biosensor lawn was inoculated with 10 µl of the compounds stock solutions deposited on sterile filter discs on top of the agar. Subsequently, another drying period was conducted to allow the antibiotics to be completely absorbed. Afterwards, the plates were incubated facing up overnight at 37 °C.

A. Full light picture after 24 h of incubation. **B.** Luminescence output was measured with a Vilber Fusion Fx documentation system equipped with a chemiluminescence filter and an exposure time of 1 minute. **C.** The table indicates the concentrations of the antibiotics used as well as their solvents. DMSO and HCl solvents were included in the plates as controls. Ethanol was previously tested (Sup. Fig. 1). Amphotericin B is included in the lower right corner as the positive control.

Ampicillin, HCl and DMSO were purchased from Carl Roth (GmbH + Co. KG, Karslruhe, Germany). Bacitracin, Chloramphenicol, Ciprofloxacin, Erythromycin, Fosfomycin, Lincomycin, Nalidixic acid, Nisin, Rifampicin and Vancomycin were purchased from Sigma-Aldrich (Merck KGaA, Darmstadt, Germany). Tetracycline was purchased from Fluka (Thermo Fisher Scientific, Waltham, Massachusetts, U.S.). The Amphotericin B solution and Kanamycin were purchased from Biowest (VWR International GmbH, Darmstadt, Germany).

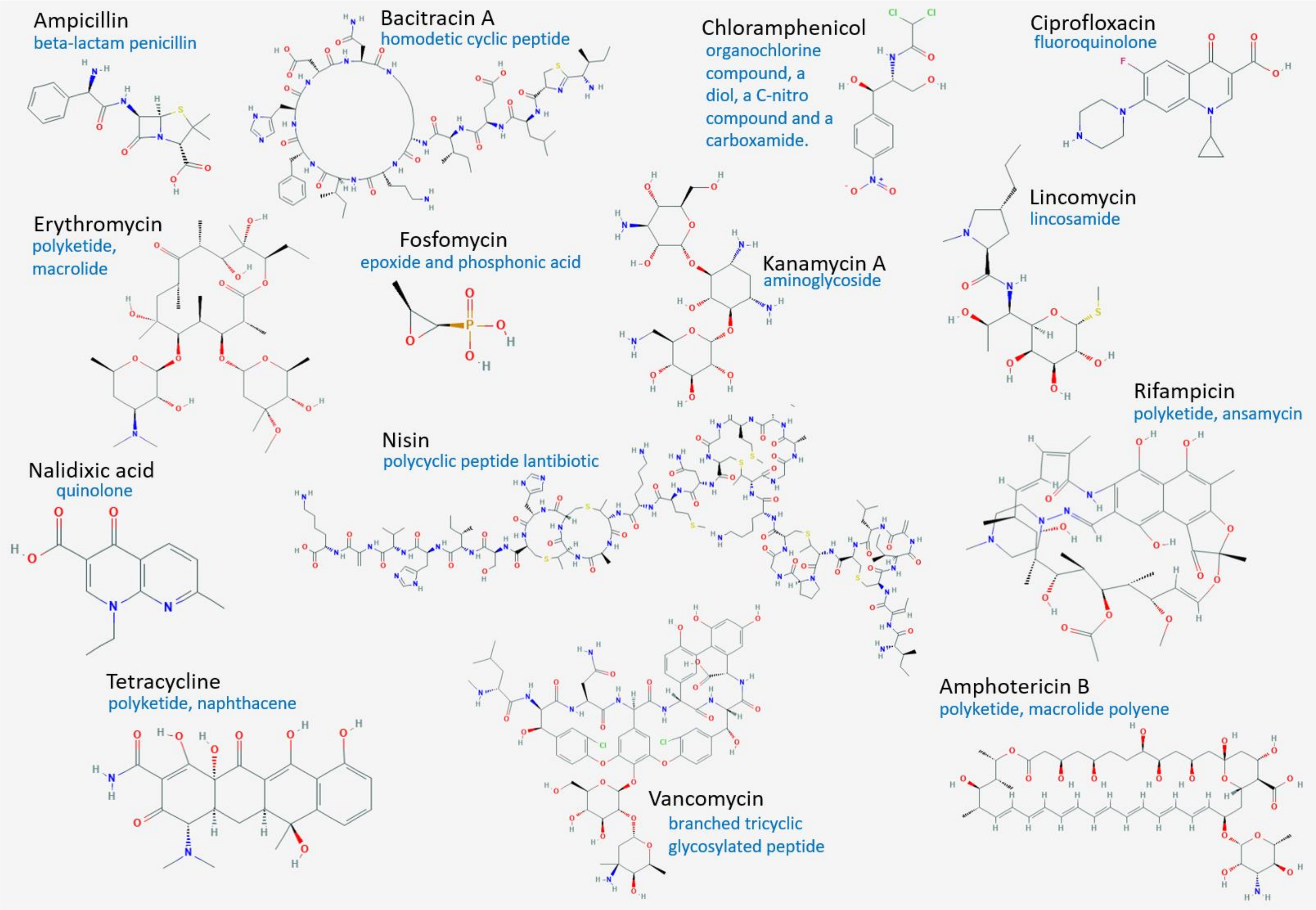
Ampicillin
beta-lactam penicillin
Bacitracin A
homodetic cyclic peptide
Chloramphenicol
organochlorine compound, a diol, a C-nitro compound and a carboxamide.
Ciprofloxacin
fluoroquinolone
Erythromycin
polyketide, macrolide
Fosfomycin
epoxide and phosphonic acid
Kanamycin A
aminoglycoside
Lincomycin
lincosamide
Rifampicin
polyketide, ansamycin
Nisin
polycyclic peptide lantibiotic
Nalidixic acid
quinolone
Tetracycline
polyketide, naphthacene
Vancomycin
branched tricyclic glycosylated peptide
Amphotericin B
polyketide, macrolide polyene

Supplementary Figure 4. Molecular structures of the non-polyene antibiotics used in the control study presented in Sup. Fig. 3. The structure of Amphotericin B is included for comparison purposes. Molecular structure images downloaded from National Center for Biotechnology Information, PubChem Database (accessed on July 13, 2020) (Kim, Thiessen et al. 2015). Structures not to scale. The PubChem Reference for Amphotericin B is included in the legend of Fig. 1 in the main text.

Ampicillin, CID=6249 (https://pubchem.ncbi.nlm.nih.gov/compound/6249#section=2D-Structure).
Bacitracin A, CID=10909430 (https://pubchem.ncbi.nlm.nih.gov/compound/10909430#section=2D-Structure).
Chloramphenicol, CID=5959 (https://pubchem.ncbi.nlm.nih.gov/compound/5959#section=2D-Structure).
Ciprofloxacin, CID=2764 (https://pubchem.ncbi.nlm.nih.gov/compound/2764#section=2D-Structure).
Erythromycin, CID=12560 (https://pubchem.ncbi.nlm.nih.gov/compound/12560#section=2D-Structure).
Fosfomycin, CID=446987 (https://pubchem.ncbi.nlm.nih.gov/compound/446987#section=2D-Structure).
Kanamycin, CID=6032 (https://pubchem.ncbi.nlm.nih.gov/compound/6032#section=2D-Structure).
Lincomycin, CID=3000540 (https://pubchem.ncbi.nlm.nih.gov/compound/3000540#section=2D-Structure).
Nalidixic acid, CID=4421 (https://pubchem.ncbi.nlm.nih.gov/compound/4421#section=2D-Structure).
Nisin, CID=16219761 (https://pubchem.ncbi.nlm.nih.gov/compound/16219761#section=2D-Structure).
Rifampicin, CID=135398735 (https://pubchem.ncbi.nlm.nih.gov/compound/135398735#section=2D-Structure).
Tetracycline, CID=54675776 (https://pubchem.ncbi.nlm.nih.gov/compound/54675776#section=2D-Structure).
Vancomycin, CID=14969 (https://pubchem.ncbi.nlm.nih.gov/compound/14969#section=2D-Structure).

Kim, S., P. A. Thiessen, et al. (2015). "PubChem Substance and Compound databases." Nucleic Acids Res 44(D1): D1202-D1213.
